# Édouard Louis´s novel The End of Eddy: A representation of hegemonic masculinity?

**DOI:** 10.12688/f1000research.151852.1

**Published:** 2024-06-10

**Authors:** Milan Mašát

**Affiliations:** 1 The Department of Czech Language and Literature, Palacky University Olomouc, Olomouc, Olomouc Region, 77140, Czech Republic

**Keywords:** The End of Eddy, narrative content analysis, hegemonic masculinity, homonationalism, socio-cultural environment, birth of the conscious homosexual, homophobia, gay, young adult literature, masculinity, coming out

## Abstract

**Background:**

In this paper we analyse the novel *The End of Eddy* by Édouard Louis. The motivation for this paper is Bourdeau’s (2020) observation that Louis’s book explores working class politics, sexuality, and masculinity.

**Methods:**

We analysed the amendment through narrative content analysis, the application of which allows us to answer the following question: Édouard Louis’s novel The End of Eddy: A representation of hegemonic masculinity?

**Results:**

We conclude that this narrative is built on contradictions that can be summarized as a conflict between a socio-cultural norm anchored in a French village and a person who does not fulfil this concept, who is outside of it. We believe that hegemonic masculinity, that is, one part of the cultural norm of a given village, causes Eddy’s inclination or consciousness of homonationalism. Thus, on the one hand, hegemonic masculinity is undoubtedly present in this novel; on the other hand, it forms a kind of background or socio-cultural environment which, although it defines itself against the given, unconsciously causes the “birth of the conscious homosexual”.

**Conclusions:**

Thus, we dare to claim that the narrative under analysis is not only a representation of hegemonic masculinity, but also an accentuation of its external and internal influence on one’s own perception of (sexual) difference.

## Introduction

The main aim of this paper is to show whether and, if so, to what extent a representation of hegemonic masculinity occurs in Édouard Louis’s novel *The End of Eddy* ( Louis, 2017). The motivation for this paper is Bourdeau’s (2020) observation that Louis’s book explores working class politics, sexuality, and masculinity. He analyses the autobiographical style of writing and the themes of marginalisation and social exclusion. However, it does not provide information about the novel’s conclusion. His paper concludes that the author emphasizes non-hegemonic masculinities in the narrative.

*Hegemonic masculinity* refers to the dominant forms of masculinity that shape social norms and expectations. These masculinities are often associated with power, control and traditional gender roles and influence men’s behaviour and perceptions in different contexts. Studies critique this concept and suggest that men’s behaviour does not always conform to a single hegemonic ideal. The portrayal of masculinity in the media and marketing, for example on product packaging, reinforces stereotypes and gender norms associated with hegemonic masculinity. Scholars have proposed alternative frameworks, such as cultural repertoires, to better capture the diversity and complexity of male experiences and expressions of masculinity beyond the traditional hegemonic model ( Nayak, 2023; Curone-Prieto, La Parra-Casado, &Vives-Cases, 2022; Berner-Rodoreda et al., 2023).

The concept of so-called hegemonic masculinity was discussed by Connell in 1995. After considerable criticism from experts, Connell revised and reintroduced it in 2005 in collaboration with Messerschmidt. In his original concept, Connell understands hegemonic masculinity as “a pattern of practices (…) that allow male dominance over women to continue. Hegemonic masculinity was separate from other masculinities, especially subordinate masculinities. (…) It was not assumed to be normal in a statistical sense, (…) but it was certainly normative. It also embodied the most cherished way of being a man.” ( Connell & Messerschmidt, 2005, p. 832). Hegemonic masculinity is simply the designation of the ideal type of man in a particular sociocultural order and historical period. Very often, hegemonic masculinity was/is associated with militarism (see Higate & Hopton, 2005). However, in addition to militarism, it is also connoted with other uniformed workers such as police officers and firefighters. Connell & Messerschmidt’s reworking of the understanding of hegemonic masculinity draws attention to two of its most important points: its plurality and its characteristic hierarchical nature. The variability and multiplicity of masculinities evident in the work is suggested, for example, by Collinson and Hearn (1996), who focus on specific masculinities associated with men’s jobs.

### Hegemonic masculinities in literature

Hegemonic masculinities in literature are deeply intertwined with power dynamics and cultural norms ( Rose, 2022 or da Silva Sousa, 2022). The concept of hegemonic masculinity highlights how masculinity is embedded in culture, normalizing male domination ( Domínguez, Campo, & Arcos, 2022). Men often face health issues due to conforming to traditional masculine roles, leading to a lack of self-care ( King et al., 2021). In literature, the normalization of hegemonic masculinity perpetuates power imbalances and justifies domination. Moreover, the intersection of hegemonic masculinity with violence in intimate relationships, including within gay couples, showcases how this construct perpetuates power dynamics through various forms of violence. Overall, literature reflects and reinforces the cultural norms and power structures associated with hegemonic masculinities, shaping societal perceptions and behaviours. Liu et al. (2022) or Green, Satyen, & Toumbourou (2024) state that *cultural norms* play a significant role in literature, influencing translation practices and the portrayal of characters. Al-Fouzan (2019) adds that translators face challenges when dealing with culture-specific references, often resorting to adaptation by deletion, replacement, or addition to bridge cultural gaps.

### Briefly about the novel *The End of Eddy*

Bourdeau (2020) or Dalibert (2018) state that *The End of Eddy* delves into themes of class, masculinity, and sexuality. The novel portrays the struggles of a young gay man from a working-class background, highlighting issues of shame, social ascension, and rejection of societal norms ( Foerster, 2016). It is situated within a broader discussion on the representation of working-class individuals in French society, shedding light on the exclusion of white working-class populations and the concept of homonationalism ( Barde & Triquenaux, 2015). *Homonationalism*, as defined by Jasbir Puar, describes a conspiracy between LGBTQ subjects or discourses of rights and nationalism, whereby some LGBTQ individuals conform to nationalist and imperialist agendas rather than being excluded ( Masri, 2022). This concept has been extended to the evaluation of the sovereignty of nations based on LGBTQ rights, leading to the term “pinkwashing” to describe nations promoting a “gay-friendly” image to distract from political violence ( Liinason, 2023). Through a blend of autobiography and fiction, Louis challenges traditional narratives and societal expectations, contributing to a larger discourse on class dynamics, gender identity, and the intersectionality of oppressions.

This article will therefore focus on the way in which the concept of hegemonic masculinity, or non-homogemonic masculinity in conjunction with homonationalism and certain cultural norms, is represented in Louis’s novel.

## Methods

The research was based on the method of narrative content analysis with the fact that you focused on the themes of hegemonic masculinity, homonationalism in the context of the accentuation of (specific) socio-cultural norms. Cornell, Brander and Peden (2023) state that narrative content analysis of literature involves an in-depth interpretation of narrative texts, especially those that relate to current events or political discourse. Qian and Sun (2022) add that it focuses on understanding the narrative structure, the presence of the narrator and the language used in the text. By studying narrative in literary works, we can discover the personal perspective of the narrators, the language they use, and the way they shape the story ( Tallarico et al., 2021). Crucially for our analysis, this method of discovery explores how narratives reflect social history, personal histories, and cultural codes, providing insight into the experiences of characters and the wider context of the narrative ( Shazad et al., 2022), which can be perceived as overarching concepts for both masculinity and homonationalism.

## Results

The publication *The End of Eddy* is based on the depiction of several contradictions. The protagonist (the author) grows up in a northern French village based purely on patriarchy. The men are seen as the breadwinners, whose concern is to provide financially for their families, for which the members are supposed to be grateful and devoted. Devotion is seen not only in terms of gratitude to parents, but also in terms of uncritical acceptance of their worldview and in following their example. Any deviation from these a priori planned and predetermined life paths is punished and punished by the entire village community. Thus Eddy, aware of his sexual difference, must struggle not only with his family and his social environment, but also with himself. The moment he realizes that he is homosexual, I under the influence of his parents, especially his mother, he dates girls and ostentatiously shows himself to them. Firstly, to avoid the bullying he experiences at school, but also to “calm public opinion” about his person, his orientation, his otherness.

Another contrast is the perception of the world by the villagers in comparison with the author’s experience of studying in a Parisian high school. Villagers consider any feminine expression (groomed appearance, cultural speech, or academic goals) in men as homosexuality, whereas in a large urban school these aspects are an integral part of life; on the contrary, their exclusion may imply a certain degree of ostracization or stigmatization of a person who does not meet these a priori notions of a Parisian.

We have already indicated that a significant part of the publication is the thematization of the relationship between men and women. Men, portrayed as the absolute rulers of families and villages, as fertilisers and as hard workers in the local factory, are emblematic of “old” France, of the old world in general. The book also highlights other socio-cultural norms by which men are judged: at the age of thirteen, Eddy’s villagers (mostly his family) are surprised that he has not yet had sexual relations with a girl. Girls who would have sexual relations with men at an inappropriate age, or who would have replaced multiple men in their search for the right one, are seen as “whores”. Women and girls are therefore in a subordinate role. And it is from this entrenched social background that Eddy breaks out. He does not represent a man who regularly gets drunk, fights, and subsequently has sex with many girls, but he is cultured and, even given his constant search for himself, rather introverted, avoiding society.

The pivotal moment that shifts Eddy’s decision-making, actions and self-perception in a certain way is when he and his friends are cornered by his mother in a garden shed during sexual play. Eddy’s mother, who still does not admit that her son could be gay, is completely surprised and passes the solution to this problem on to Eddy’s father. He resolves the situation in the typical social manner: he slaps Eddy and forbids him from the activities in question. At this point - although Eddy tries to change his sexual orientation - he plans his escape, deciding definitively not to go to high school, where all the citizens of the village went and still go, but to continue his studies in Paris. He thus sets himself against the established order: on the one hand, he inadvertently gives evidence of his minority sexual orientation, but also distances himself from the assumed following of village traditions: graduating from the local secondary school and then joining the neighbouring factory. Although Eddy and his family try to keep what happened in their shed a secret from everyone. However, the situation comes to light when one of the direct participants explodes everything at the school. All the ridicule and certain forms of persecution are directed at Eddy because he has been breaking with the conventions and norms of his life so far.

## Discussion

We believe that the above, in our opinion, the driving moments of Louis’s narrative, in a way represent the thematic levels we have pursued in the story. Homonationalism is portrayal of heteronomous masculinity is put in the context of village cultural norms that are - metaphorically speaking - passed down from generation to generation and whose disruption is unforgivable. These village cultural norms are based on the role of the male - the breadwinner, the tough guy - who has control over the events of the entire social group. In the context of this role, it is understood that men are representatives of heteronormativity (hegemonic masculinity), that is, their other social role is to procreate the events to which they can transmit the norms anchored in the village and punish them at their discretion for their violation. Homonationalism is explicated indirectly in the book. Eddy tries to be part of the majority - heteronormative - society. His homonationalism only becomes apparent after he is discovered in a shed with his friends - this moment can be seen as a turning point, when he realises that he is a member of the homosexual minority and that he wants to be part of it in Paris.

## Conclusion

In the introduction of the paper, we asked the question *Édouard Louis’s novel The End of Eddy: A representation of hegemonic masculinity?* The answer to this question is not clear-cut. We are of the opinion that hegemonic masculinity, that is, one part of the cultural norm of the village in question, causes Eddy’s inclination or awareness of homonationalism. Thus, on the one hand, hegemonic masculinity is undoubtedly represented in this novel, on the other hand, it forms a kind of backdrop or socio-cultural environment which, although it is defined against the given, unconsciously causes the “birth of the conscious homosexual”. Thus, we dare to state that the analyzed narrative is not only a representation of hegemonic masculinity, but also an accentuation of its influence external and internal to one’s own perception of (sexual) difference.

### Ethics and consent

Ethical approval and consent were not required.
