# Édouard Louis´s novel The End of Eddy: A representation of hegemonic masculinity?

**DOI:** 10.12688/f1000research.151852.2

**Published:** 2024-09-13

**Authors:** Milan Mašát

**Affiliations:** 1 The Department of Czech Language and Literature, Palacky University Olomouc, Olomouc, Olomouc Region, 77140, Czech Republic

**Keywords:** The End of Eddy, narrative content analysis, hegemonic masculinity, homonationalism, socio-cultural environment, birth of the conscious homosexual, homophobia, gay, young adult literature, masculinity, coming out

## Abstract

**Background:**

In this paper we analyse the novel *The End of Eddy* by Édouard Louis. The motivation for this paper is Bourdeau’s (2020) observation that Louis’s book explores working class politics, sexuality, and masculinity.

**Methods:**

We analysed the amendment through narrative content analysis, the application of which allows us to answer the following question: Édouard Louis’s novel The End of Eddy: A representation of hegemonic masculinity?

**Results:**

We conclude that this narrative is built on contradictions that can be summarized as a conflict between a socio-cultural norm anchored in a French village and a person who does not fulfil this concept, who is outside of it. We believe that hegemonic masculinity, that is, one part of the cultural norm of a given village, causes Eddy’s inclination or consciousness of homonationalism. Thus, on the one hand, hegemonic masculinity is undoubtedly present in this novel; on the other hand, it forms a kind of background or socio-cultural environment which, although it defines itself against the given, unconsciously causes the “birth of the conscious homosexual”.

**Conclusions:**

Thus, we dare to claim that the narrative under analysis is not only a representation of hegemonic masculinity, but also an accentuation of its external and internal influence on one’s own perception of (sexual) difference.

## Introduction

The main aim of this paper is to show whether and, if so, to what extent a representation of hegemonic masculinity occurs in Édouard Louis’s novel *The End of Eddy* ( Louis, 2017). The motivation for this paper is Bourdeau’s (2020) observation that Louis’s book explores working class politics, sexuality, and masculinity. Drawing on Bourdeau’s insights, we analyse the autobiographical style of Louise’s writing and the themes of marginalisation and social exclusion implemented in this publication, seeking to substantiate or refute Bourdea’s claim that Louise emphasises non-hegemonic masculinities in this novel. We perceive non-hegemonic masculinities as a violation of the principles and procedures of hegemonic masculinity, i.e. a certain way of violation or transformation in the field of gender studies and the hierarchy of various societies.

*Hegemonic masculinity* refers to the dominant forms of masculinity that shape social norms and expectations. These masculinities are often associated with power, control and traditional gender roles and influence men’s behaviour and perceptions in different contexts. Studies critique this concept and suggest that men’s behaviour does not always conform to a single hegemonic ideal. The portrayal of masculinity in the media and marketing, for example on product packaging, reinforces stereotypes and gender norms associated with hegemonic masculinity. Scholars have proposed alternative frameworks, such as cultural repertoires, to better capture the diversity and complexity of male experiences and expressions of masculinity beyond the traditional hegemonic model ( Nayak, 2023; Curone-Prieto, La Parra-Casado, &Vives-Cases, 2022; Berner-Rodoreda et al., 2023).

The concept of so-called hegemonic masculinity was discussed by Connell in 1995. After considerable criticism from experts, Connell revised and reintroduced it in 2005 in collaboration with Messerschmidt. In his original concept, Connell understands hegemonic masculinity as “a pattern of practices (…) that allow male dominance over women to continue. Hegemonic masculinity was separate from other masculinities, especially subordinate masculinities. (…) It was not assumed to be normal in a statistical sense, (…) but it was certainly normative. It also embodied the most cherished way of being a man.” ( Connell & Messerschmidt, 2005, p. 832). Hegemonic masculinity is simply the designation of the ideal type of man in a particular sociocultural order and historical period. Very often, hegemonic masculinity was/is associated with militarism (see Higate & Hopton, 2005). However, in addition to militarism, it is also connoted with other uniformed workers such as police officers and firefighters. Connell & Messerschmidt’s reworking of the understanding of hegemonic masculinity draws attention to two of its most important points: its plurality and its characteristic hierarchical nature. The variability and multiplicity of masculinities evident in the work is suggested, for example, by Collinson and Hearn (1996), who focus on specific masculinities associated with men’s jobs. Swidler (1986) points out that these alternative models of culture, with all its implications, are a kind of base that continues to evolve. Hirsch and Kachtan (2018) underlines the need to look at this concept in a more multi-layered way, even though the basis of this theory largely corresponds to cultural practice. Johri (2023) states that Connell’s concept of hegemonic masculinity explores how dominant groups establish authority over gender identities, legitimizing patriarchy and subordinating women and men embodying subordinate masculinity. Yang (2020) underlines that this concept of hegemonic masculinity explores the legitimation and complexities beyond traditional notions, shaping influential perspectives in feminist sociology. Based on these statements we will try to point out the application of this concept in the analysed novel.

### Hegemonic masculinities in literature

Hegemonic masculinities in literature are deeply intertwined with power dynamics and cultural norms ( Rose, 2022 or da Silva Sousa, 2022). The concept of hegemonic masculinity highlights how masculinity is embedded in culture, normalizing male domination ( Domínguez, Campo, & Arcos, 2022). Men often face health issues due to conforming to traditional masculine roles, leading to a lack of self-care ( King et al., 2021). In literature, the normalization of hegemonic masculinity perpetuates power imbalances and justifies domination. Moreover, the intersection of hegemonic masculinity with violence in intimate relationships, including within gay couples, showcases how this construct perpetuates power dynamics through various forms of violence. Overall, literature reflects and reinforces the cultural norms and power structures associated with hegemonic masculinities, shaping societal perceptions and behaviours. Liu et al. (2022) or Green, Satyen, and Toumbourou (2024) state that *cultural norms* play a significant role in literature, influencing translation practices and the portrayal of characters. Al-Fouzan (2019) adds that translators face challenges when dealing with culture-specific references, often resorting to adaptation by deletion, replacement, or addition to bridge cultural gaps.

We are aware that we apply the relevant concept to the analysis without its other nuances (e.g. the fact that this concept can be implemented in different ways). We chose this procedure to simplify the text and to point out a specific application of one possible interpretation of this concept.

### Briefly about the novel *The End of Eddy*

Bourdeau (2020) or Dalibert (2018) state that *The End of Eddy* delves into themes of class, masculinity, and sexuality. The novel portrays the struggles of a young gay man from a working-class background, highlighting issues of shame, social ascension, and rejection of societal norms ( Foerster, 2016). It is situated within a broader discussion on the representation of working-class individuals in French society, shedding light on the exclusion of white working-class populations and the concept of homonationalism ( Barde & Triquenaux, 2015). *Homonationalism*, as defined by Jasbir Puar, describes a conspiracy between LGBTQ subjects or discourses of rights and nationalism, whereby some LGBTQ individuals conform to nationalist and imperialist agendas rather than being excluded ( Masri, 2022). This concept has been extended to the evaluation of the sovereignty of nations based on LGBTQ rights, leading to the term “pinkwashing” to describe nations promoting a “gay-friendly” image to distract from political violence ( Liinason, 2023). Through a blend of autobiography and fiction, Louis challenges traditional narratives and societal expectations, contributing to a larger discourse on class dynamics, gender identity, and the intersectionality of oppressions.

This article will therefore focus on the way in which the concept of hegemonic masculinity, or non-homogemonic masculinity and certain cultural norms, is represented in Louis’s novel. We are aware that the concept of homonacism is not primarily emphasized in the publication, but there are moments, implicit or explicit, that refer to this concept. Thus, although we will refer to this concept, and in most cases in conjunction with non-homogenous masculinity, we are aware that this is not a representation of the central idea of the narrative.

## Methods

The research was based on the method of narrative analysis with the fact that we focused on the themes of hegemonic masculinity in the context of the accentuation of (specific) socio-cultural norms. Cornell, Brander and Peden (2023) state that narrative content analysis of literature involves an in-depth interpretation of narrative texts, especially those that relate to current events or political discourse. Qian and Sun (2022) add that it focuses on understanding the narrative structure, the presence of the narrator and the language used in the text. By studying narrative in literary works, we can discover the personal perspective of the narrators, the language they use, and the way they shape the story ( Tallarico et al., 2021). Crucially for our analysis, this method of discovery explores how narratives reflect social history, personal histories, and cultural codes, providing insight into the experiences of characters and the wider context of the narrative ( Shazad et al., 2022), which can be perceived as overarching concepts for both masculinity and homonationalism.

## Results

The publication *The End of Eddy* is based on the depiction of several contradictions. We are not afraid of using the term *contradiction* because we want to draw a somewhat harsh attention to certain aspects of the novel that fill the semantic field of the term. The protagonist (the narrator) grows up in a northern French village based especially on patriarchy. The men are seen as the breadwinners, whose concern is to provide financially for their families, for which the members are supposed to be grateful and devoted. Devotion is seen not only in terms of gratitude to parents, but also in terms of uncritical acceptance of their worldview and in following their example. Any deviation from these a priori planned and predetermined life paths is punished by the entire village community. Thus Eddy, aware of his sexual difference, must struggle not only with his family and his social environment, but also with himself. The moment he realizes that he is homosexual, I under the influence of his parents, especially his mother, he dates girls and ostentatiously shows himself to them. Firstly, to avoid the bullying he experiences at school, but also to “calm public opinion” about his person, his orientation, his otherness. Thus, although there is a necessary coming to terms with his otherness, Eddy is also praised. However, it can be observed that he is praised mainly for his upbringing, which is a credit to his immediate environment, not his own.

Another contrast is the perception of the world by the villagers in comparison with the author’s experience of studying in an Amiens high school. Villagers consider any feminine expression (groomed appearance, cultural speech, or academic goals) in men as homosexuality, whereas in a large urban school these aspects are an integral part of life; on the contrary, their exclusion may imply a certain degree of ostracization or stigmatization of a person who does not meet these a priori notions of an Amiens. We believe that the narrative’s relatively explicit portrayal of the boys’ attitudes to parenting and behaviour is, to some extent, exaggerated in order to clearly highlight the differences in the relevant area and Eddy’s perception of them.

We have already indicated that a significant part of the publication is the thematization of the relationship between men and women. Men, portrayed as the absolute rulers of families and villages, as fertilisers and as hard workers in the local factory, are emblematic of “old” France, of the old world in general. The book also highlights other socio-cultural norms by which men are judged: at the age of thirteen, Eddy’s villagers (mostly his family) are surprised that he has not yet had sexual relations with a girl. Girls who would have sexual relations with men at an inappropriate age, or who would have replaced multiple men in their search for the right one, are seen as “whores”. Women and girls are therefore in a subordinate role. And it is from this entrenched social background that Eddy breaks out. He does not represent a man who regularly gets drunk, fights, and subsequently has sex with many girls, but he is cultured and, even given his constant search for himself, rather introverted, avoiding society.

The pivotal moment that shifts Eddy’s decision-making, actions and self-perception in a certain way is when he and his friends are cornered by his mother in a garden shed during sexual play. Eddy’s mother, who still does not admit that her son could be gay, is completely surprised and passes the solution to this problem on to Eddy’s father. He resolves the situation in the typical social manner: he slaps Eddy and forbids him from the activities in question. At this point - although Eddy tries to change his sexual orientation - he plans his escape, deciding definitively not to go to high school, where all the citizens of the village went and still go, but to continue his studies in Paris. He thus sets himself against the established order: on the one hand, he inadvertently gives evidence of his minority sexual orientation, but also distances himself from the assumed following of village traditions: graduating from the local secondary school and then joining the neighbouring factory. Although Eddy and his family try to keep what happened in their shed a secret from everyone. However, the situation comes to light when one of the direct participants explodes everything at the school. All the ridicule and certain forms of persecution are directed at Eddy because he has been breaking with the conventions and norms of his life so far.

## Discussion

We believe that the above, in our opinion, the driving moments of Louis’s narrative, in a way represent the thematic levels we have pursued in the story. Homonationalism is portrayal of heteronomous masculinity is put in the context of village cultural norms that are - metaphorically speaking - passed down from generation to generation and whose disruption is unforgivable. To put it plainly - this is the transmission of certain norms and, in a way, stereotypes from generation to generation, especially in connection with the gender of persons. These village cultural norms are based on the role of the male - the breadwinner, the tough guy - who has control over the events of the entire social group. In the context of this role, it is understood that men are representatives of heteronormativity (hegemonic masculinity), that is, their other social role is to procreate the events to which they can transmit the norms anchored in the village and punish them at their discretion for their violation. Homonationalism is explicated indirectly in the book. Eddy tries to be part of the majority - heteronormative - society. His homonationalism only becomes apparent after he is discovered with his friends in the shed - this moment can be considered a turning point, when he realizes that he is a member of the homosexual minority and that he wants to be part of it while attending high school.

## Conclusion

At the beginning of the article, we asked the question Édouard Louis’ novel The End of Eddy: A Representation of hegemonic masculinity? The answer to this question is not clear-cut. We are of the opinion that hegemonic masculinity, i.e. one part of the cultural norm of the given village, causes Eddy’s inclination or awareness of homonationalism, at the level of self-discovery and subsequent acceptance, which culminates in Eddy’s desire to blend in with the LGBTQ community while studying in high school. On the one hand, hegemonic masculinity is undoubtedly represented in this novel, on the other hand, it forms a kind of scenery or socio-cultural environment, which, although it is defined in relation to the given, unconsciously causes “the birth of the conscious homosexual”. We therefore dare to claim that the analysed narrative is not only a representation of hegemonic masculinity, but also an accentuation of its external and internal influence on one’s own perception of (sexual) difference. We realize that we are not giving a clear answer to the question that was the starting point of this article. Aspects of subordinate, complicit, and protest/alternative masculinities can certainly be traced in the novel, but we are of the opinion that these elements of masculinity are (un) implicitly subordinated precisely to the accentuation of hegemonic masculinity in the novel, the requirement of which on the basis of anchored social norms led to Eddy’s homonationalism on the level an awareness of belonging to a minority sexual orientation and an effort to perceive and present this belonging as a positive difference from the social norm or the majority.

### Ethics and consent

Ethical approval and consent were not required.
