# Supplementary material for: Édouard Louis´s novel The End of Eddy: A representation of hegemonic masculinity?
Source: F1000Res. 2024 Sep 13;13:612. Originally published 2024 Jun 10. [Version 2] doi: 10.12688/f1000research.151852.2 (PMC11971620; doi:10.12688/f1000research.151852.2)
Supplement: Peer review history

# Reviewer report: Reviewer response for version 2

**DOI:** 10.5256/f1000research.171805.r370371

**Published:** 2025-04-04

**Authors:** E Ngestirosa Endang Woro Kasih

**Affiliations:** 1 Universitas Teknokrat Indonesia, Lampung, Indonesia

The article **"Édouard Louis’s novel The End of Eddy: A Representation of Hegemonic Masculinity?"** explores the portrayal of masculinity, sexuality, and socio-cultural norms in Louis’s novel through narrative content analysis.

 The study examines how hegemonic masculinity—dominant, traditional male roles—shapes the protagonist Eddy’s self-awareness and eventual identification with the LGBTQ community. The novel highlights the rigid expectations placed on men in a working-class French village and how deviations from these norms lead to social punishment. The article argues that while hegemonic masculinity is strongly represented in the novel, it also acts as a catalyst for Eddy’s realization of his sexual identity, leading to his conscious embrace of his difference. The study further discusses the intersection of masculinity with homo nationalism, though this aspect remains a secondary theme in the novel.

**Is the work clearly and accurately presented, and does it cite the current literature?**

**Partly** – The article is well-structured and references relevant literature on hegemonic masculinity, gender norms, and homonationalism. However, some parts of the analysis are complex and could be more clearly articulated, particularly in linking theoretical frameworks directly to textual evidence from the novel.

**​​​Is the study design appropriate and is the work technically sound?**

**Yes** – The use of narrative content analysis is appropriate for examining themes of masculinity in literature. The theoretical background is well-established, and the study aligns with established research methodologies.

**Are sufficient details of methods and analysis provided to allow replication by others?**

**Partly** – While the study provides a general overview of its approach, more specific details about how the narrative content analysis was conducted (e.g., coding process, selection criteria for themes) would improve replicability.

**If applicable, is the statistical analysis and its interpretation appropriate?**

**Not applicable** – The study is qualitative and does not involve statistical analysis.

**Are all the source data underlying the results available to ensure full reproducibility?**

**No source data required** – Since the study is based on literary analysis rather than empirical data collection, raw data is not necessary for reproducibility.

**Are the conclusions drawn adequately supported by the results?**

**Partly** – The conclusions effectively summarize the key findings, particularly in discussing the role of hegemonic masculinity in shaping Eddy’s identity. However, the argument about homo nationalism could be further substantiated with more direct textual evidence from the novel to avoid speculation.

 The article is **well-researched and cites relevant literature**, including Connell’s concept of hegemonic masculinity and its revisions. However, **some parts of the argument could be more clearly articulated**, particularly in connecting theoretical frameworks directly to textual evidence.

 The **study design is appropriate**, using narrative content analysis, but **the methodology could be explained in greater detail to improve replicability**—for instance, specifying how themes were identified in the text.

 The **conclusions are largely supported by the analysis**, though the discussion of homo nationalism would benefit from stronger textual evidence. Since the study is qualitative, **no statistical analysis is required**, and **source data is not necessary for reproducibility**.

 Overall, the article presents a compelling exploration of masculinity in *The End of Eddy*, though greater clarity in methodology and argumentation would enhance its impact.

Is the work clearly and accurately presented and does it cite the current literature?

Partly

If applicable, is the statistical analysis and its interpretation appropriate?

Not applicable

Are all the source data underlying the results available to ensure full reproducibility?

No source data required

Is the study design appropriate and is the work technically sound?

Yes

Are the conclusions drawn adequately supported by the results?

Partly

Are sufficient details of methods and analysis provided to allow replication by others?

Partly

Reviewer Expertise:

My research focuses on American literature and minority literature, including works by women, children, and various indigenous tribes. Additionally, I explore literary theories such as deconstruction, popular literature, and other relevant theoretical frameworks.

I confirm that I have read this submission and believe that I have an appropriate level of expertise to confirm that it is of an acceptable scientific standard, however I have significant reservations, as outlined above.

# Reviewer report: Reviewer response for version 2

**DOI:** 10.5256/f1000research.171805.r323379

**Published:** 2024-09-18

**Authors:** Robert Morrell

**Affiliations:** 1 Centre for Higher Education Development, University of Cape Town, Mowbray, Western Cape, South Africa

I am happy for it to be indexed without further revision.

Is the work clearly and accurately presented and does it cite the current literature?

Partly

If applicable, is the statistical analysis and its interpretation appropriate?

Not applicable

Are all the source data underlying the results available to ensure full reproducibility?

No source data required

Is the study design appropriate and is the work technically sound?

Partly

Are the conclusions drawn adequately supported by the results?

Partly

Are sufficient details of methods and analysis provided to allow replication by others?

Yes

Reviewer Expertise:

Gender, Masculinities, Violence, Fatherhood, Schooling, HIV interventions, Southern Theory, University staff development, Southern African History

I confirm that I have read this submission and believe that I have an appropriate level of expertise to confirm that it is of an acceptable scientific standard.

# Reviewer report: Reviewer response for version 1

**DOI:** 10.5256/f1000research.166539.r311523

**Published:** 2024-09-10

**Authors:** Astrid Berner-Rodoreda

**Affiliations:** 1 Heidelberg Institute of Global Health, University of Heidelberg, Heidelberg, Baden-Württemberg, Germany

**REVIEW**

 The author asks the interesting question whether the autobiographical novel by Edouard Louis is a representation of hegemonic masculinity. The question requires a critical engagement with Connell’s concept of hegemonic masculinity and an analysis of the novel in this regard.

 While this paper provides some interesting thoughts, it is perhaps a bit too brief to delve into the concept of hegemonic masculinities vis-à-vis the novel in sufficient depth and detail.

**Introduction**

 In the first paragraph, it remains unclear if the author is interested in analysing Louis’ book or Bourdeau’s interpretation of it. The “it” in the second last sentence has no clear reference.

 The paragraphs on hegemonic masculinity could have gone into slightly more detail on the concept and the various critiques of it. Hegemonic masculinity has played an overarching role in masculinity studies of various academic disciplines over a number of decades and allowed scholars to analyse the dynamics of a given society in upholding and reinforcing certain aspects of masculinity as the “most honoured way of being a man” [ 1 ]. However, the concept has also been critiqued for gender stereotyping [ 2 ], for being vague in referring to social practice, a masculine ideal or both [ 3 ], [ 4 ] and for encompassing a plurality of contradictory ideals [ 5 ].

 For the concept of cultural repertoires, I would recommend to add the following references [ 4 ], [ 6 ] .

 The author brings in a second concept in the introduction, that of homonationalism. The rationale for utilizing this concept in analysing Louis’ book does not become altogether clear as the story of Eddie is a rather localized story of the author’s experience of growing up, struggling and eventually embracing his sexual orientation in a close-knit village. Villagers seemed to largely endorse and uphold concepts of men as strong, working-class, main income-earners, heterosexually virile, alcohol-drinking with a certain acceptance of violence (yet tolerance towards violent men seemed to differ considerably in the community). The book does not speak about France or French society as such - to interpret the book through the concept of homonationalism seems therefore a little far-fetched to me. There was little in the book to suggest any embracing of nationalist ideas by the LGBTQ+ community (which does not figure in the book as a community). The book alludes to more tolerance towards different sexual orientations at the high school/boarding school in Amiens. The fear of strangers (e.g., people of colour) was mentioned from the father’s perspective, not from an LGBTQ+ perspective. The reason for using this concept for analysing the book remains obscure to me.

**Methods**

 Perhaps the author could reference the “narrative content analysis” method -  the reference provided refers to a content analysis (counting themes) in a narrative review. I would recommend to explain what a “narrative content analysis” entails and what themes were counted. My impression is that a narrative analysis rather than a content analysis was employed.

 The first sentence sounds a bit odd – who is the “you”?

**Results**

 I would guard against sweeping statements, such as “The protagonist (the author) grows up in a northern French village based *purely* on patriarchy” or “Any deviation from these a priori planned and predetermined life paths is punished and punished by the entire village community”. This, to me, goes beyond what we can gather from the book. In fact, the book is more nuanced. Some mothers congratulate Eddy’s mother for bringing up such a nice boy. This section could be improved by providing more nuance.

 I would also reconsider where to apply the term “contradiction” – is a homosexual boy growing up in a conservative village environment a “contradiction”? Is perhaps not rather the behaviour of people in the village (family and friends, school-mates) contradictory - on the one hand upholding heterosexual norms, yet also engaging in homosexual acts (friends); bullying and beating Eddy, but also acknowledging and celebrating his performing talents (school mates); the father threatening Eddy to never practise homosexuality again, yet protecting Eddy from his elder brother who wants to kill Eddy; the father letting himself be beaten up by his older son so that Eddy will survive; both parents enabling him to pursue secondary education with a baccalauréat when none of their other children seem to be given the same opportunity (parents).

 In the French version of Louis’ book, he did not go to Paris but to a secondary school in Amiens, a town in Northern France. Not a lot of detail is provided about this time – I feel the second paragraph in the results section is a bit of an over-interpretation of these pages.

 The next paragraph brings out the contrast between the expectations towards men and women quite well. Here one could have delved a bit deeper into the analysis of a hierarchy of masculinities and femininities. Again, adding nuances and seeing where the book fits neatly into the hegemonic masculinity framework and where there is tension might add more to our understanding. The mother could work until she earned more than her husband, then she was told to quit her job; yet in other families, the mother was a teacher or a single mother –  the degree to which women could make their own decisions thus seemed to vary across the village and according to their socio-economic situation. Eddy’s girlfriend Laura had a bad reputation in the village and was called a “pute” or “salope” (whore/slut) which emphasizes the point the author makes about different societal expectations towards the sexual behaviour of men and women. At the same time, going out with her increased Eddy’s respect by his male cousin – Eddy describes it as a form of admiration which motivates him to keep going with this relationship. In terms of masculinities, we see Eddy’s father being strictly against homosexuality and making right wing political statements in that regard but he protects a homosexual man when the mob turned against this man. Eddy’s father also never laid hands on his wife or children and would rather accept to be beaten up by his son than to retaliate, yet would often beat other men if they seemed interested in his wife. The father became unemployed, yet was able to still maintain the drinking habits with his friends who seemed to provide the alcohol for him. There seem to be many layers of masculinity that could be explored in more depth in the novel.

**Discussion**

 My recommendation would be to discuss the extent to which the hegemonic masculinity framework is upheld through the novel – where do we see hegemonic, subordinate, marginalized masculinities – how are femininities portrayed?

 I fail to see what this novel has to do with homonationalism – where in this story is there a favourable association between the LGBTQ+ community and nationalist ideology? Eddy himself takes on his father’s fear of people of colour when he goes to Amiens – this is the only point that might be interpreted as homonationalism in my opinion, and this would be on rather thin grounds. I also do not understand the sentence: “Homonationalism is portrayal of heteronomous masculinity is put in the context of village cultural norms that are - metaphorically speaking - passed down from generation to generation and whose disruption is unforgivable”.  Another sentence also needs rewriting: “their other social role is to procreate the events to which they can transmit the norms anchored in the village and punish them at their discretion for their violation”.

**Conclusion**

 The link of this novel to homonationalism remains obscure. I would drop this concept altogether and summarize here where the hegemonic framework is helpful and where the story may not support the framework. I think this would help the reader gain a deeper understanding of the complexity of masculinities and femininities in this autobiographical novel.

Is the work clearly and accurately presented and does it cite the current literature?

Partly

If applicable, is the statistical analysis and its interpretation appropriate?

Not applicable

Are all the source data underlying the results available to ensure full reproducibility?

Yes

Is the study design appropriate and is the work technically sound?

Partly

Are the conclusions drawn adequately supported by the results?

Partly

Are sufficient details of methods and analysis provided to allow replication by others?

Partly

Reviewer Expertise:

masculinity; stigma; qualitative research methods; policy; Global Health; HIV interventions; COVID-19

I confirm that I have read this submission and believe that I have an appropriate level of expertise to confirm that it is of an acceptable scientific standard, however I have significant reservations, as outlined above.

## References

[1] : Hegemonic Masculinity. *Gender & Society* .2005;19(6) : 10.1177/0891243205278639 829-859 10.1177/0891243205278639

[2] : Masculinities, Crime and Criminology. *Sage* .1998; Reference source

[3] : Rethinking Hegemonic Masculinity in a Globalizing World. *Men and Masculinities* .2008;11(1) : 10.1177/1097184X08315102 86-103 10.1177/1097184X08315102

[4] : Is “Hegemonic Masculinity” Hegemonicas Masculinity? Two Israeli Case Studies. *Men and Masculinities* .2018;21(5) : 10.1177/1097184X17696186 687-708 10.1177/1097184X17696186

[5] : Cultural Repertoires and Situated Selections as an Alternative Framework to Hegemonic Masculinities: Findings From Eswatini. *Am J Mens Health* .2023;17(1) : 10.1177/15579883231152110 15579883231152110 10.1177/15579883231152110 36823951 PMC9969463

[6] : Culture in Action: Symbols and Strategies. *American Sociological Review* .1986;51(2) : 10.2307/2095521 10.2307/2095521

# Response

**Published:** 2024-09-11

**Authors:** Milan Mašát

**Affiliations:** Czech Language and Literature, Palacky University Olomouc, Olomouc, Olomouc Region, Czech Republic

Dear Astrid Berner-Rodoreda,

 Thank you very much for the very stimulating comments on my article, which I tried to incorporate into the text(even taking into account the second review). All the changes I made are highlighted in red in the text.

 Sincerely,

 Milan Mašát

# Reviewer report: Reviewer response for version 1

**DOI:** 10.5256/f1000research.166539.r295721

**Published:** 2024-08-02

**Authors:** Robert Morrell

**Affiliations:** 1 Centre for Higher Education Development, University of Cape Town, Mowbray, Western Cape, South Africa

This is an interesting, sophisticated but somewhat dense paper with a very specific focus explained as “Bourdeau’s (2020) observation that Louis’s book explores working class politics, sexuality, and masculinity”. It seeks to use the concept, hegemonic masculinity, developed by Connell (and later debated and refined over decades) to analyse the gendered representation of masculinity by asking (in the title) the question: “A representation of hegemonic masculinity?” I wonder whether this was a helpful framing because, as my notes below indicate, I think this question encourages a binaried approach which in the end produces a static analysis where it would have been possible, drawing on other concepts in Connell’s masculinities conceptual toolkit, to offer a more nuanced analysis.

 In my notes below, I provide examples to support my conclusion but also note stylistic infelicities which, if addressed, would make the paper easier for readers.

**Methods: “**We analysed the amendment through narrative content analysis,”

Comment: It is not clear what ‘amendment’ refers to. Needs clarification.

**Introduction**

**“** *Hegemonic masculinity* refers to the dominant forms of masculinity that shape social norms and expectations. These masculinities are often associated with power, control and traditional gender roles and influence men’s behaviour and perceptions in different contexts.”

 Comment: Although the author follows this definition with a review of a literature on hegemonic masculinity, my sense is that this initial offering is too bland and doesn’t do justice to the contested nature of the concept. The author uses hegemonic masculinity as an equivalent for dominant masculinity and indeed other authors adopt this approach as well. But in fact Connell explicitly distinguishes hegemony from dominance with the emphasis in the case, following Gramsci, of hegemony on agreement/consent rather than force (which is associated with dominance). [ ref1 ]; [ ref 2 ]; [ ref 3 ].

 But I have to concede that the concept is widely used and remains useful in analysis and authors have to take some theoretical risks when they use it.

**Hegemonic masculinities in literature**

**Comment**: This section fixes the content of masculinity as oppressive which is not in line with the contested nature of hegemonic masculinity. In other words, the author is assuming a fixed content of hegemonic masculinity rather than allowing a more fluid use of the concept where the content and form of hegemonic masculinity are contested and dynamic.

 “Overall, literature reflects and reinforces the cultural norms and power structures associated with hegemonic masculinities, shaping societal perceptions and behaviours.”

**Comment**: Here we see the effects of adopting a narrow definition of hegemonic masculinity. This statement effectively is reproductionist where literature (no matter its provenance, politics and so on) is simply an arrow in the quiver of hegemonic masculinity. It is difficult to see how any literature, in terms of this statement, can be counter-hegemonic or how it can be part of a process of envisioning alternative (or protest) masculinities.

 “non-homogemonic masculinity”

 This is confusing for the reader – what does this mean? It isn’t defined or explained.

**Methods**

**Comment**: It is not clear whether the ‘narrator’ refers to the voice of a character in the novel or actually refers to the novelist himself. This confusion is compounded in the Results section by the statement “The protagonist (the author)”.

**Results**: “based purely on patriarchy”.

**Comment**: The intrusion of concepts from gender studies or the social sciences is necessary but this occurs often in a static way where the content and purpose of the term is assumed which gives the impression that life is one-dimensional and uncontested. I think one can use a concept like patriarchy without reifying it and I would have thought that literature would be an ideal place for such nuanced attention.

 The description of Eddy’s life in his village is clear and well-written and the choices he makes are well contextualised.

**Discussion:**

 “Homonationalism is portrayal of heteronomous masculinity is put in the context of village cultural norms that are - metaphorically speaking - passed down from generation to generation and whose disruption is unforgivable”.

**Comment**: I find this a difficult sentence to follow.

**Conclusion**:

**Comment**: The author, having asked the question, offers an ambiguous answer. Yes and No. I am not sure that this enhances our understanding of the gendered significance of this novel. Would it not have been better to embrace the other elements of Connell’s masculinities framework – subordinate, complicit and protest/alternative masculinities to show how these are present in the novel? This would also have allowed the author to evade what appears to be a binaried treatment of masculinity – either hegemonic or not hegemonic. My concerns at the outset were that the key concepts have been rendered in static ways and I think that the conclusion reflects this limitation.

Is the work clearly and accurately presented and does it cite the current literature?

Partly

If applicable, is the statistical analysis and its interpretation appropriate?

Not applicable

Are all the source data underlying the results available to ensure full reproducibility?

No source data required

Is the study design appropriate and is the work technically sound?

Partly

Are the conclusions drawn adequately supported by the results?

Partly

Are sufficient details of methods and analysis provided to allow replication by others?

Yes

Reviewer Expertise:

Gender, Masculinities, Violence, Fatherhood, Schooling, HIV interventions, Southern Theory, University staff development, Southern African History

I confirm that I have read this submission and believe that I have an appropriate level of expertise to confirm that it is of an acceptable scientific standard, however I have significant reservations, as outlined above.

## References

[1] : Negotiating the Field of Masculinity. *Men and Masculinities* .2009;12(1) : 10.1177/1097184X07309502 30-44 10.1177/1097184X07309502

[2] : Engendering Gendered Knowledge. *Men and Masculinities* .2012;15(1) : 10.1177/1097184X11428384 56-76 10.1177/1097184X11428384

[3] : The Salience of “Hegemonic Masculinity”. *Men and Masculinities* .2019;22(1) : 10.1177/1097184X18805555 85-91 10.1177/1097184X18805555

# Response

**Published:** 2024-09-11

**Authors:** Milan Mašát

**Affiliations:** Czech Language and Literature, Palacky University Olomouc, Olomouc, Olomouc Region, Czech Republic

Dear Robert Morrell,

 thank you very much for the very stimulating comments on my article, which I tried to incorporate into the text (even taking into account the second review). All the changes I made are highlighted in red in the text.

 Sincerely,

 Milan Mašát
